# Supplementary material for: Antimony 3: Extending human-readable model definitions for SBML Level 3 Core and Packages
Source: PLoS Comput Biol. 2026 Oct 12;22(10):e1014207. doi: 10.1371/journal.pcbi.1014207 (PMC13671645; doi:10.1371/journal.pcbi.1014207)
Supplement: Supplementary file 1 [file pcbi.1014207.s001.pdf]

Antimony Runtime Evaluation

To evaluate Antimony's runtime efficiency, we tested its conversion runtime with models from BioModels [1]. We selected the ten largest curated models in BioModels for this test. For each model, we passed the SBML string as input to Antimony and measured the wall-clock time required to convert it to an Antimony string. We then used the resulting Antimony string as input and measured the wall-clock time required to convert it back to an SBML string. We repeated each conversion 10 times for every selected model. All measurements were performed on an Apple M3 Mac with an 8-core CPU (4 performance and 4 efficiency cores) and 16 GB of RAM, running macOS 15.1.1. The results are shown in A. As shown in this figure, the runtime for both SBML-to-Antimony and Antimony-to-SBML conversions remained within a reasonable range across all tested models, including the largest curated model in BioModels, `BIOMD0000001063`.

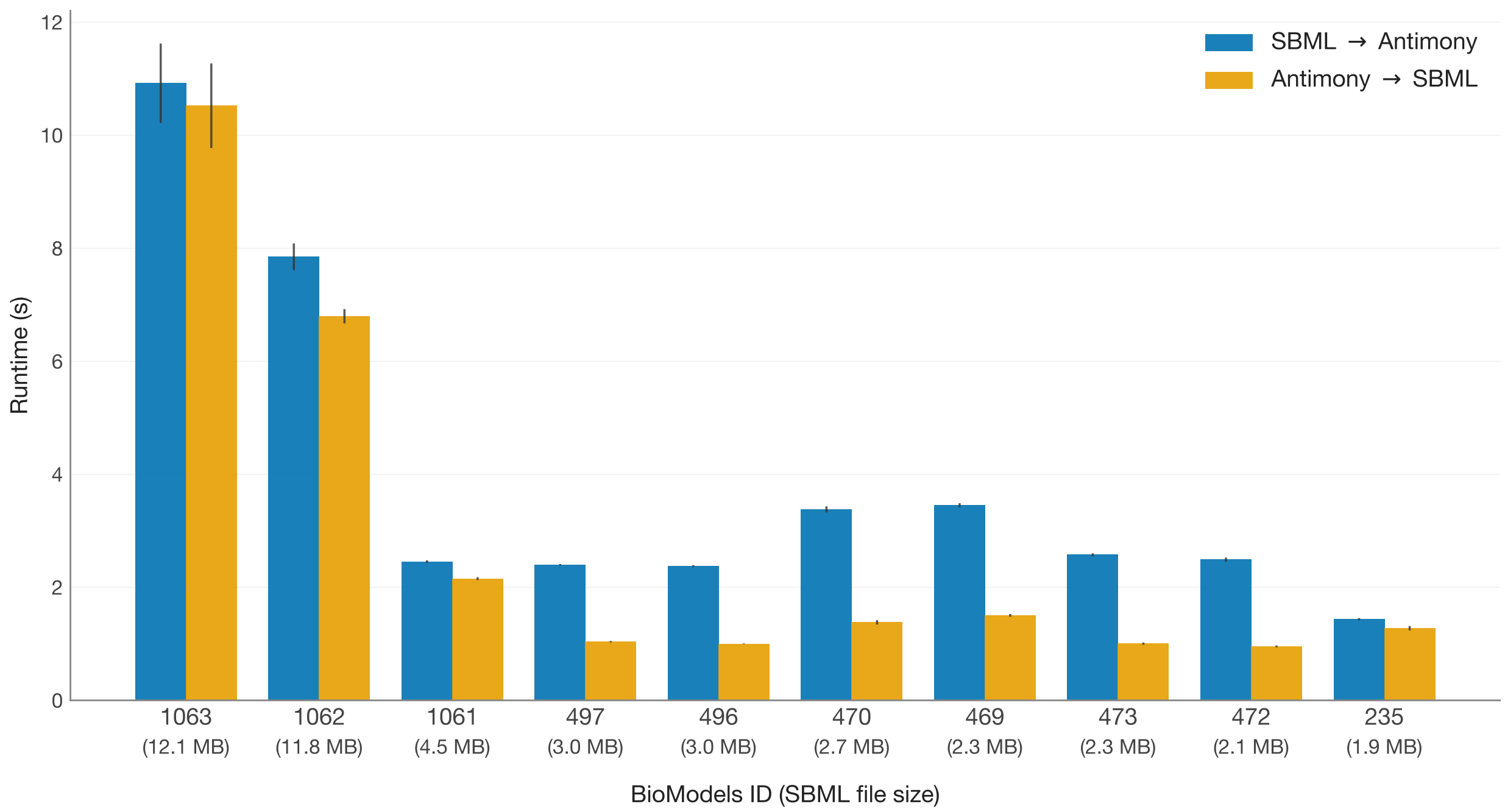

Fig A. **Antimony runtime for SBML-to-Antimony and Antimony-to-SBML conversions.** The test includes the ten largest curated models in BioModels (selected based on SBML file size). The BioModels identifier and SBML file size are shown for each model. Bars show the mean runtime over 10 repetitions, and error bars indicate the 95% confidence interval.

References

[1] Rahuman S. Malik-Sheriff, Mihai Glont, Tung V. N. Nguyen, et al. Biomodels—15 years of sharing computational models in life science. *Nucleic Acids Research*, 48(D1):D407–D415, 2020.
